# Shenmai injection enhances cisplatin-induced apoptosis through regulation of Mfn2-dependent mitochondrial dynamics in lung adenocarcinoma A549/DDP cells

**DOI:** 10.20517/cdr.2021.94

**Published:** 2021-12-10

**Authors:** Yushi Chen, Ye Sun, Qiuyu Zhao, Chunying Liu, Chun Wang

**Affiliations:** ^1^College of Integrated Chinese and Western Medical, Liaoning University of Traditional Chinese Medicine, Shenyang 110847, Liaoning, China.; ^2^Basic Medical College, Shenyang Medical College, Shenyang 110034, Liaoning, China.; ^3^Key Laboratory of Ministry of Education for TCM Viscera-State Theory and Applications, Liaoning University of Traditional Chinese Medicine, Shenyang 110847, Liaoning, China.

**Keywords:** SMI, NSCLC, cisplatin-resistant, Mfn2, mitochondrial dynamics, apoptosis

## Abstract

**Aim: **Chemoresistance is the biggest obstacle in cancer treatment. Our previous study demonstrated that Shenmai injection (SMI), a Chinese herbal medicine, enhanced the antitumor effect of cisplatin via glucose metabolism reprogramming. This study aimed to further determine whether the SMI sensitizes the non-small cell lung cancer (NSCLC) cells to cisplatin through regulation mitochondrial dynamics.

**Methods: **The Kaplan-Meier Plotter database was used to investigate the relationship between mRNA expression of mitofusin-2 (Mfn2) and the survival analysis of NSCLC patients. The protein expression of Mfn2 in a lung adenocarcinoma tissue chip was detected by immunohistochemistry staining. The expression of Mfn2 and ATAD3A were compared between cisplatin-sensitive A549 and cisplatin-resistant A549/DDP cells. Additionally, A549/DDP cells were co-treated with cisplatin and SMI to detect mitochondrial morphology by fluorescent staining, apoptosis-related protein expression with Western blotting, and mitochondrial membrane potential (ΔΨm) with flow cytometry analysis.

**Results: **The mean survival time of the Mfn2^low^ group was significantly lower than that of the Mfn2^high^ group by Kaplan-Meier Plotter database analysis, and the Mfn2 protein expression level was lower in cancer tissues than in adjacent tissues. The combination of SMI and cisplatin induced dynamic changes in A549/DDP cells, with increased mitochondrial fusion followed by upregulation of Mfn2 and downregulation of ATAD3A and reduced mitochondrial mass and ΔΨm. Moreover, SMI significantly enhanced cisplatin-induced A549/DDP apoptosis, upregulated Bax and the active subunit of caspase-3, and downregulated Bcl-2 expression, as shown via Hoechst staining and flow cytometry analysis.

**Conclusion: **Our findings suggest SMI enhances cisplatin-induced apoptosis through regulation of Mfn2-dependent mitochondrial dynamics in cisplatin-resistant lung adenocarcinoma cells.

## INTRODUCTION

Lung cancer is one of the most common malignancies worldwide and has become the leading cause of cancer-related mortality in China. Non-small cell lung cancer (NSCLC) accounts for approximately 80%-85% of all lung cancers^[1]^. Cisplatin-based combination chemotherapy is the standard drug treatment for NSCLC; however, multidrug resistance to chemotherapy results in failure of NSCLC treatment^[2]^.

Herbal and traditional medicines [traditional Chinese medicine (TCM)] are important complementary and alternative medicines used for developing anticancer therapies in Asia. Shenmai injection (SMI), the water-soluble extract of *Radix Ginseng Rubra* and *Radix Ophiopogonis*, is derived from a well-known traditional Chinese formula, Shendong Yin, and approved by the China Food and Drug Administration as an injectable treatment^[3]^. SMI is widely applied in cardiovascular diseases, such as myocardial infarction, arrhythmia, and myocardial fibrosis^[4-6]^. Current clinical practice has shown that SMI is a valid alternative medicine for anti-tumor therapy and alleviates chemotherapy-induced side effects in NSCLC, breast cancer, and pancreatic cancer^[7]^. Additionally, experimental studies have demonstrated the mechanism of reversal of adriamycin and paclitaxel in colorectal cancers^[8]^, as well as enhancing the antitumor effect of cisplatin via glucose metabolism reprogramming in lung adenocarcinoma A549/DDP cells as detected by our previous study^[9]^.

Recent studies have brought to light the fundamental role of mitochondria in the resistance to chemotherapy. Mitochondria are highly dynamic organelles, and their morphology and function are strictly preserved by the balance between mitochondrial fission and fusion^[10]^. The ﬁssion and fusion processes are generically termed mitochondrial dynamics. Mitochondrial ﬁssion is mediated by GTPase dynamin-related protein-1 (Drp1), fission protein 1 (Fis1), and mitochondrial fission factor. The fusion process is regulated by two membranes, the mitofusin proteins (Mfn1 and Mfn2) and the optic atrophy 1 protein (Opa1)^[11]^. We reported the antitumor mechanisms of various TCMs as drug resistance reversal agents. Shenqi Fuzheng injection effectively improved cisplatin sensitivity in human lung adenocarcinoma A549/DDP cells through Mfn2-mediated cell cycle arrest and apoptosis. Bu-Zhong-Yi-Qi decoction reversed cisplatin resistance by inducing mitochondrial autophagy in A549/DDP cells^[12,13]^. In the present study, we focused on Mfn2-mediated mitochondrial dynamics and continued to explore the possible role of SMI in reversing cisplatin resistance in NSCLC cells.

## METHODS

### Cell culture and chemicals

The A549 human lung adenocarcinoma cell line and the A549/DDP variant cell line were obtained from the National Infrastructure of Cell Line Resource, Beijing, China. Cells were cultured in RPMI1640 medium (Hyclone, USA) supplemented with 10% fetal bovine serum (Hyclone, USA) and grown at 37 °C in a 5% CO_2_ atmosphere. As in our previous report, the A549/DDP cell medium contained 16.7 μM cisplatin to maintain drug resistance, with no cisplatin (Sigma, USA) for two weeks before the experiment^[9]^. SMI containing 200 mg *Radix Ginseng Rubra *and 200 mg *Radix Ophiopogonis*/1 mL was purchased from the Hebei Shenwei Pharmaceutical Factory.

### Patient survival assay

We investigated the correlation between Mfn2 mRNA levels with overall survival using the online survival analysis tool Kaplan-Meier Plotter (http://kmplot.com/analysis/). Briefly, Mfn2 was fed into the database, and the survival time (months) of patients with lung adenocarcinoma was analyzed according to the hazard ratio (HR), 95% confidence interval (CI), and log-rank *P*-value shown in the main panel of the operating system. According to the expression of Mfn2 mRNA, 305 lung adenocarcinoma cases in the database were ranked from low expression to high expression. Cases were divided into a high expression group and a low expression group^[14]^.

### Human tissue immunohistochemistry

The lung adenocarcinoma tissue microarray (HLugA150CS02), including lung adenocarcinoma and paired adjacent normal lung tissue (*n* = 75), was obtained from Outdo Biotech Co. Ltd. (Shanghai, China). The rights for the microarray for scientific research was approved by the Committees for the Ethical Review of Research at the Taizhou Hospital, Zhejiang province. The expression level of the Mfn2 protein in the tissue microarray was detected using tissue immunohistochemistry (IHC). IHC staining of the microarray was performed as previously described. Briefly, after dewaxing, rehydration, and blocking of endogenous peroxidase, the microarray was incubated with anti-Mfn2 and then a secondary antibody, stained with DAB, and images were captured using a tissue chip scanner (Pannoramic MIDI, Hungary)^[15]^. The TMA software of the Quant center analysis software was applied to automatically identify and analyze the percentage of cells in each tissue point, and the staining score was comprehensively determined according to the weighted results of the percentage of positively stained cells (1 point, ≤ 25% positive cells; 2 points, 26%-49%; 3 points, 50%-74%; 4 points, ≥ 75%) and the staining intensity (0 point, negative; 1 point, weak; 2 points, moderate; 3 points, strong). Using the median score as the critical value, we defined the samples with a score less than or equal to the median as the low-expression group and those with a score above the median as the high-expression group^[16]^.

### Chemo-resistance and assessment of cell viability

The viability of A549/DDP cells following treatment with SMI or co-treatment with cisplatin and SMI was determined via CCK-8 assay. A549/DDP cells were seeded into four 96-well plates at a density of 1 × 10^4^/well, and then the cells were incubated with different concentrations of SMI (5, 10, 20, 40, 80, or 160 mg/mL) for 24 h at 37 °C. CCK-8 kit reagent was added into each well, and the optical density was detected at 460 nm using the Multiscan Spectrum (BioTek, ELx800, USA). For the co-treatment of cisplatin and SMI, the cells were re-treated with IC_5_, IC_10_, and IC_20_ of SMI (13, 20, and 30 mg/mL, respectively) for 2 h and then different concentrations of cisplatin (15, 30, 60, 120, and 240 μM) for another 24 h incubation^[9]^. Then, chemoresistance was tested using the above treatment scheme. The IC_20_ of cisplatin was calculated using the cell survival curve formula previously published in the literature^[13]^.

### Western blot analysis

A549 cells or A549/DDP cells were seeded, and the proteins were extracted, quantified, and transferred to a polyvinylidene fluoride membrane. Mfn2, ATAD3A (Abcam, Cambridge, UK), and β-actin antibodies (CST, Boston, USA) were used. The proteins were visualized using ECL reagent (Vazyme, Nanjing, China), and the results were measured using ImageJ software (National Institutes of Health, Bethesda, MD, USA). Additionally, a WES™ machine (ProteinSimple, San Jose, CA, USA) was used for apoptosis-related protein detection. A549/DDP cells were retreated with SMI at a dose of 13, 20, and 30 mg/mL for 2 h and then incubated with cisplatin for another 24 h. During sample loading, the whole-cell protein samples, sealing solution, anti-Bax, anti-Bcl-2, anti-Cleaved-caspase 3, secondary antibody (ABclonal, Wuhan, China), luminescent solution, and eluent were successively added to the plate according to a set 3 h run procedure^[17]^. Data were analyzed using Compass for SW software.

### Hoechst staining

Morphological changes in chromatin were characteristic of apoptosis, which was assessed by Hoechst 33342 (Sigma, USA). After treatment with cisplatin and SMI, A549/DDP cells were stained with Hoechst, and the results were captured by a fluorescence microscope (ZEISS, Observer AL, Germany)^[18]^.

### Annexin V-FITC/PI double staining

After treatment with cisplatin and SMI, A549/DDP cells were stained using an Annexin V-FITC/PI assay kit (Vazyme, Nanjing, China) according to the manufacturer’s instructions. The results were measured via a flow cytometer (BD Biosciences, San Diego, CA) and analyzed with BD Accuri C6 Software^[19]^.

### Mitochondrial labeling and mitochondrial mass analysis

After treatment with cisplatin and SMI, A549/DDP cells were stained with 20 nM Mito-Tracker Green (Beyotime, Shanghai, China) according to the manufacturer’s instructions and detected and harvested using confocal microscopy (Olympus, FV10i, Japan). Mitochondrial mass was analyzed by calculating the fluorescent intensity with ImageJ software^[20]^.

### Mitochondrial membrane potential assay

Mitochondrial potential (ΔΨm) was determined using fluorescent dye Rhodamine 123 (Beyotime, Shanghai, China). After treatment with cisplatin and SMI, A549/DDP cells were stained with Rhodamine 123 and analyzed via a flow cytometer (Beckman Coulter, California, US)^[21]^.

### Statistical analysis

Each experiment was performed separately at least three times. The data are expressed as means ± SD. Statistical analyses were performed using SPSS 22.0 and GraphPad Prism software version 7.0. The survival curve was constructed using the Kaplan-Meier method and compared by log-rank test. Pearson chi-square test or Student’s *t*-test was used to detect the relationship between Mfn2 expression and clinical pathological factors in the IHC results. Statistical comparisons between different groups were evaluated using one-way ANOVA, and *P *< 0.05 was considered a statistically significant difference.

## RESULTS

### Mfn2 is a promising biomarker for the prognosis of NSCLC

**Figure 1:**
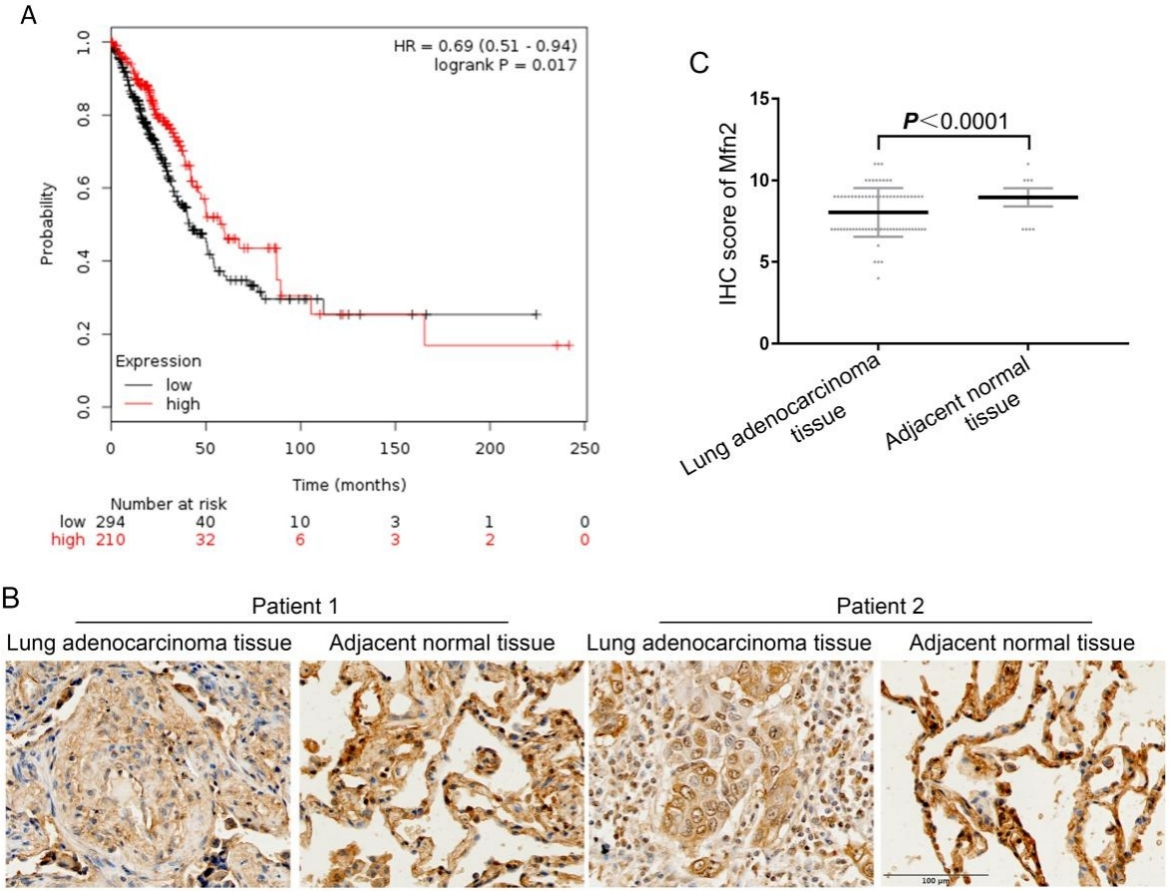
Analysis of clinical data on Mfn2 in lung adenocarcinoma patients: (A) the relationship between mRNA expression of *Mfn2* and survival rate as determined via the Kaplan-Meier Plotter database; and (B, C) IHC staining and score of Mfn2 protein expression, *n* = 75. IHC: Immunohistochemistry.

The effects of the mitochondrial fusion protein Mfn2 in tumors are currently unclear. We first investigated the relationship between Mfn2 mRNA expression and the survival of NSCLC patients using the Kaplan-Meier Plotter database. The analysis showed that the mean survival of the Mfn2^low^ group (*n* = 210) was 41.17 months, which was significantly lower than that in the Mfn2^high^ group (*n* = 294), which was 59.67 months (HR = 0.69, 95%CI: 0.51-0.94, *P* < 0.05) [Figure 1A]. Next, we examined Mfn2 protein expression in 75 pairs of human lung adenocarcinoma tissue microarrays via IHC staining, and the results show that Mfn2 expression level in lung adenocarcinoma was lower than in the matched paracancerous tissues (*P* < 0.001) [Figure 1B and C]. Furthermore, we assessed the association of Mfn2 expression with lung adenocarcinoma clinical features. The results show no significant statistical difference in gender, age, tumor size, or TNM stage between the high Mfn2 expression group and the low Mfn2 expression group [Table 1].

**Table 1:** The relationship between Mfn2 expression and clinical variables in lung adenocarcinoma patients as determined by IHC staining

**Variable**	**Mfn2 expression**		***P*** ** value**
	**High**	**Low**	
Sample size	47	28	
Gender			
Female	22 (46.8%)	13 (46.4%)	0.975
Male	25 (53.2%)	15 (53.6%)	
Age			
≥ 60	28 (59.6%)	17 (60.7%)	0.922
< 60	19 (40.4%)	11 (39.3%)	
Tumor size, cm			
< 5	32 (68.0%)	23 (82.1%)	0.183
≥ 5	15 (32.0%)	5 (17.9%)	
		
TNM stage			
I-II	35 (74.5%)	24 (85.7%)	0.250
III-IV	12 (25.5%)	4 (14.3%)	

IHC: Immunohistochemistry.

### Mfn2 expression was low in cisplatin-resistant lung adenocarcinoma cells

**Figure 2:**
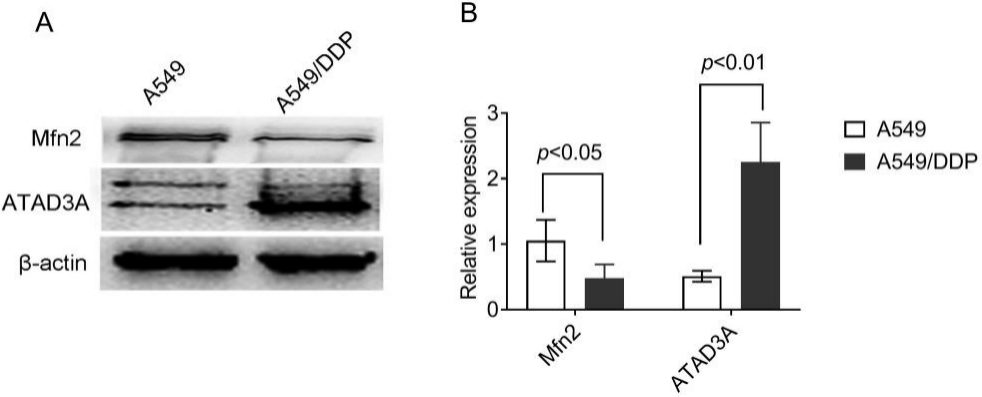
The expression levels of Mfn2 and ATAD3A in A549 and A549/DDP cells as determined by Western blot analysis: (A) the results of Western blot; and (B) the quantified analysis by densitometry. The results are shown as the mean ± standard deviation, *n* = 3.

To determine whether Mfn2 is related to chemical resistance in NSCLC, we compared the expression level of Mfn2 in cisplatin-sensitive lung adenocarcinoma A549 cells and cisplatin-resistant A549/DDP cells. The results show that the expression of Mfn2 was significantly lower in A549/DDP cells compared to A549 cells [Figure 2], which has been affirmed in previous studies^[12]^.

The ATAD3A protein, an important number of the ATPase family AAA domain-containing protein 3 (ATAD3), is not only involved in mitochondrial dynamics but also takes part in cell death modulation^[22]^. We compared the expression of ATAD3A in cisplatin-sensitive A549 cells and cisplatin-resistant A549/DDP cells. Contrary to the expression of Mfn2, the expression levels of ATAD3A were upregulated in A549/DDP *vs*. A549 cells [Figure 2].

### SMI significantly enhanced cisplatin-induced A549/DDP cell cytotoxicity

**Figure 3:**
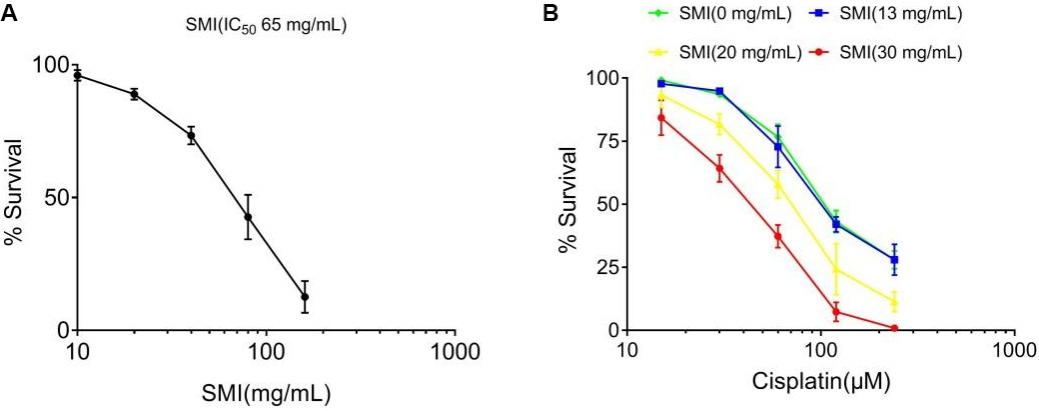
SMI reversed cisplatin resistance in A549/DDP cells. (A) Direct cytotoxic effects of SMI in A549/DDP cells. A549/DDP cells were treated with various concentrations (5, 10, 20, 40, 80, and 160 mg/mL) of SMI for 24 h. (B) Cytotoxic effects of SMI and cisplatin in A549/DDP cells. A549/DDP cells were pre-treated with various concentrations (13, 20, and 30 mg/mL) of SMI (the approximate IC_5_, IC_10_, and IC_20_, respectively) for 2 h and then exposed to cisplatin (15, 30, 60, 120, and 240 μM) for another 24 h. The cell viability was determined using a Cell Counting Kit. Each data point represents the mean ± SD of the results from four individual measurements. SMI: Shenmai injection.

To determine the cytotoxic effects of SMI, we treated A549/DDP cells with various concentrations (5, 10, 20, 40, 80, and 160 mg/mL) of SMI. The results show that SMI exhibited direct antitumor effects (the IC_5_, IC_10_, and IC_20 _values were 13, 20, and 30 mg/mL, respectively) [Figure 3A]. Next, we incubated A549/DDP cells with different concentrations of SMI and cisplatin (15, 30, 60, 120, and 240 μM) collectively. The results show that SMI significantly enhanced cisplatin-induced cytotoxicity [Figure 3B].

### SMI enhanced cisplatin-induced cytotoxicity by modulating Mfn2-dependent mitochondrial dynamics

**Figure 4:**
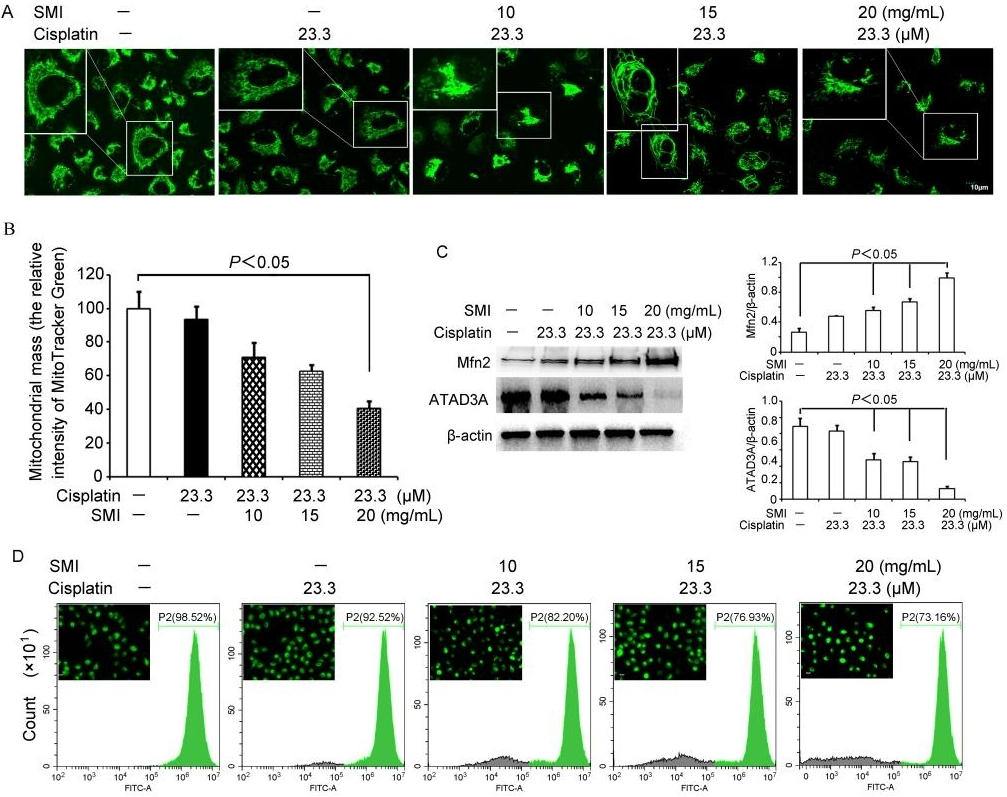
The combination of SMI and cisplatin induced Mfn2-dependent mitochondrial dynamic changes in A549/DDP cells. A549/DDP cells were pre-treated with various concentrations (13, 20, and 30 mg/mL) of SMI for 2 h and then exposed to cisplatin (25 μM) for another 24 h. (A) A549/DDP cells were labeled with the MitoTracker Green probe. The cells in the SMI and cisplatin treatment groups displayed mitochondrial spheres or ovals of widely different size, some several times larger than in the untreated cells’ mitochondrial tubules, as shown by a confocal laser scanning microscope. (B) Decrease in mitochondrial mass as determined by statistical analysis of the MitoTracker Green fluorescence intensity in (A) by ImageJ. (C) Western blotting detection and the quantified analysis by densitometry. Mean ± standard deviation, *n* = 3. (D) Decrease of ΔΨm in A549/DDP cells treated with a combination of SMI and cisplatin. Cells were dyed via Rhodamine 123, and the peak of fluorescence intensity of the SMI and cisplatin co-treatment groups shifted left as determined by flow cytometry and fluorescence microscopy (400×). SMI: Shenmai injection; ΔΨm: mitochondrial potential.

Mitochondrial dynamics have been implicated in tumorigenesis^[23]^. We labeled mitochondria with a MitoTracker Green probe and observed the results via a confocal laser scanning microscope. The mitochondria of the cisplatin alone and cisplatin + SMI groups were thread-like and filamentous and distributed in the perinuclear space, especially in the cisplatin + SMI groups, when compared with the cisplatin alone group [Figure 4A]. In addition, mitochondrial mass of the A549/DDP cells was decreased, and the greatest effect was shown in the cisplatin (23.3 μM, equivalent to IC_20_^[9]^) + SMI (30 mg/mL) group compared with the cisplatin (-) + SMI (-) group (*P* < 0.05) [Figure 4B]. We also detected the change in expression of Mfn2 and ATAD3A, and the results show that Mfn2 gradually increased and ATAD3A expression decreased, following the mitochondrial morphology alterations [Figure 4C]. ΔΨm is another assessment indicator for mitochondrial morphology that is equally important to apoptosis. Figure 4D shows that cisplatin combined with SMI notably attenuated mitochondrial membrane potential.

### A combination of SMI and cisplatin induced apoptosis in human lung adenocarcinoma A549/DDP cells

**Figure 5:**
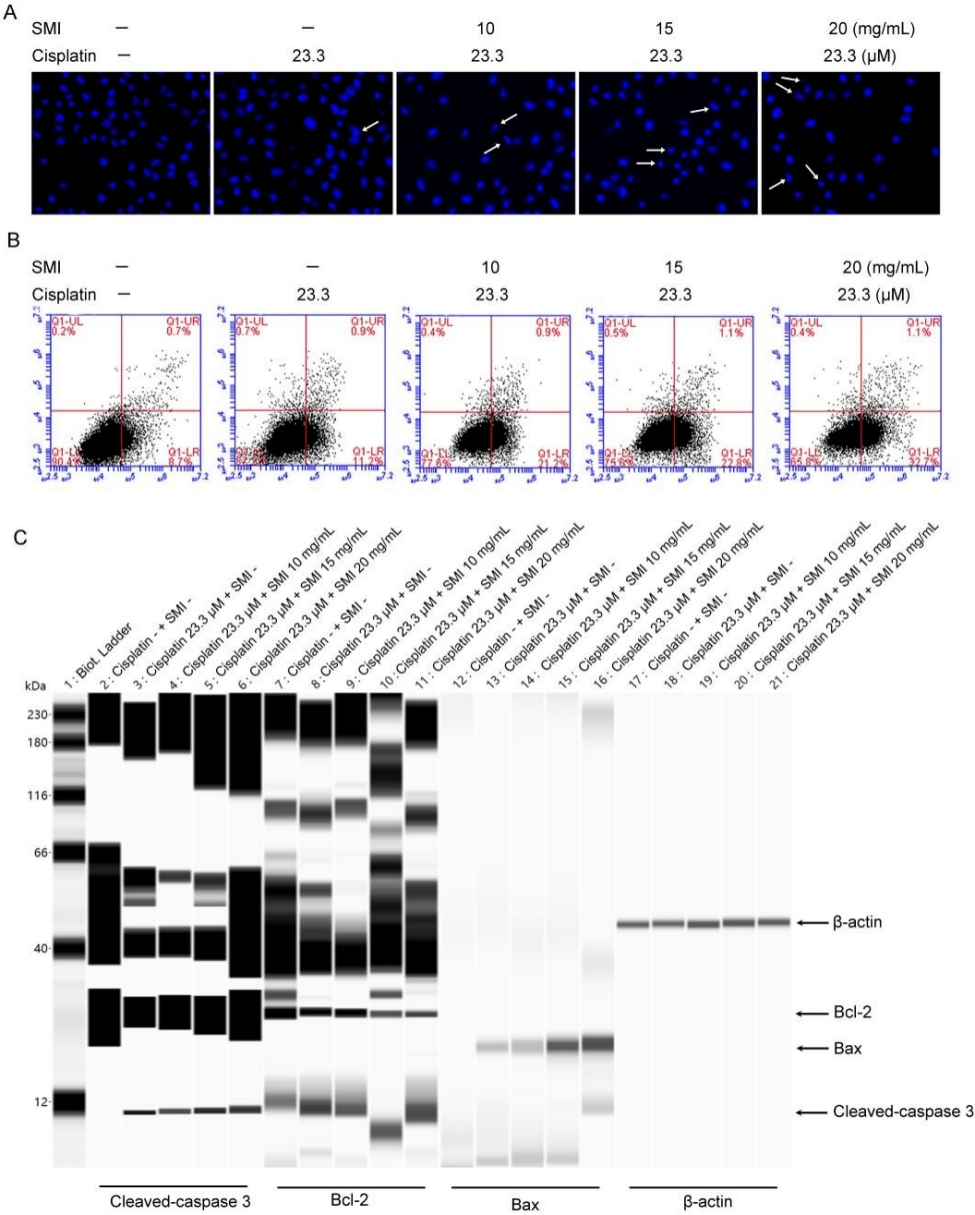
Combination of SMI and cisplatin induced apoptosis in human lung adenocarcinoma A549/DDP cells. (A) Apoptosis detection by Hoechst staining. White arrows show apoptotic cells with nuclear condensation (400×). (B) Cells were dyed by Annexin V-FITC/PI and detected by flow cytometry. (C) Simple Western blot detection for apoptosis-related proteins (Bcl-2, Bax, and cleaved-caspase 3). SMI: Shenmai injection.

We further investigated the relationship between mitochondrial dynamic changes and cell death in A549/DDP cells co-treated with SMI and cisplatin. Hoechst staining was conducted for initial apoptosis detection, and the results show that apoptotic A549/DDP cells increased gradually with the combination of cisplatin and increasing SMI treatment [Figure 5A]. Then, apoptosis was further confirmed by flow cytometry using Annexin V-FITC and PI double staining. The results show that apoptotic A549/DDP cells (in the Q1-UR quadrant and the Q1-LR quadrant) increased from 12.1% in the cisplatin (23.3 μM) group to 22.1% in the cisplatin (23.3 μM) + SMI (10 mg/mL) group, 23.9% in the cisplatin (23.3 μM) + SMI (15 mg/mL) group, and 33.8% in the cisplatin (23.3 μM) + SMI (20 mg/mL) group [Figure 5B]. We further investigated the expression of Bcl-2 and Bax and the initiation of caspase-3, the three important effectors of apoptosis, in A549/DDP cells by Simple Western blot analysis. The results show declining expression of the anti-apoptotic Bcl-2 protein and increasing expression of the apoptosis-promoting Bax protein in the groups treated with the combination of cisplatin and increasing concentrations of SMI. In addition, cleaved-caspase 3, the active subunit of the mature caspase-3, was significantly increased in the A549/DDP cells incubated with cisplatin and SMI compared to the cisplatin alone group [Figure 5C].

## DISCUSSION

At present, radiotherapy and chemotherapy remain the first comprehensive treatment choices for preventing recurrence following surgery. Cisplatin-based chemotherapy is widely used in the clinical treatment of NSCLC. However, the growing problem of cisplatin resistance remains an important factor leading to multidrug resistance in tumor cells, failure of clinical treatment, and ultimately the death of patients^[24]^.

Recently, Mfn2 has attracted much attention in cancer studies, but its exact role is not well established. In some studies, Mfn2 has been demonstrated to be an oncogenic gene, whereas others have identified antioncogenic effects^[25]^. We investigated the relationship between Mfn2 mRNA expression and clinical characteristics using the Kaplan-Meier Plotter database. The results confirm that Mfn2 may play an antioncogenic role; patients with high Mfn2 expression had a better prognosis than those with lower expression. To further clarify the role of Mfn2 in NSCLC, we compared the Mfn2 expression levels in 75 pairs of lung adenocarcinoma and matched paracancerous tissues. The results show that the expression level of Mfn2 in lung adenocarcinoma was lower than that in matched paracancerous tissues. Until now, few studies have focused on the role of Mfn2 in lung adenocarcinoma, some of which presented results inconsistent with our study showing that Mfn2 was downregulated in human lung adenocarcinoma tissues and Mfn2 exhibited antitumor activity *in vivo* and *in vitro*^[26]^. In addition, in our study, we did not identify a correlation between Mfn2 expression and clinical pathological parameters such as tumor size and TNM stage. This point is consistent with one of the studies, in which there was similarly no significant correlation between Mfn2 expression and TNM stage, although Mfn2 expression was higher in lung adenocarcinoma tissues than in adjacent tissues^[27]^.

Similarly, there are opposing reports on the effects of Mfn2 in cell and animal studies. One study showed that Mfn2 knockdown deregulated cell proliferation, cell cycle modulation, and invasion in cancer cells^[28]^. However, increasing data indicate that Mfn2 exhibits an anti-tumor role by inhibiting cancer cell proliferation, migration, and invasion *in vitro* and decreasing tumor progression *in vivo*^[29-32]^. Overexpression of Mfn2 triggered cervical tumor cell apoptosis via a mitochondrial-dependent pathway; repressed breast cancer cell proliferation, migration, and invasion by the inhibiting Ras-ERK1/2 signaling pathway; and inhibited urinary bladder carcinoma cell proliferation via inhibition of G_1_ to S phase transition^[31,32]^. However, studies on the effect of Mfn2 on chemotherapy resistance in cancer are very limited. One study reported that the expression of Mfn2 is upregulated in cisplatin-resistant murine leukemia L1210/DDP cells compared with the parental L1210 cells^[33]^. On the contrary, our previous study found that Mfn2 expression in cisplatin-resistant lung adenocarcinoma A549/DDP cells was lower than cisplatin-susceptible A549 cells. In addition, Mfn2 expression in A549/DDP cells continued to decrease when treated with the IC_10_ concentration of cisplatin^[12]^. Here, we re-confirmed that Mfn2 expression in A549/DDP cells was significantly lower than that in A549 cells. The difference of Mfn2 expression between the article referenced above and ours is likely due to the different roles of Mfn2 in different types or species of target cells. Further study is needed to verify this.

ATAD3 is a mitochondrial membrane-bound ATPase expressed in many organisms that plays an important role in mitochondrial DNA replication and transcription^[34]^. ATAD3 is localized to the mitochondrial inner membrane, and the topology of ATAD3 shows that it interacts with both the inner and outer mitochondrial membranes^[35]^. ATAD3A is reportedly involved in mitochondrial dynamics, mitochondrial activity, cholesterol metabolism, and cell growth and cancer. Furthermore, ATAD3A is confirmed as an anti-tumor cytokine involved in sensitivity to chemotherapy and radiation in most cancers^[23,36]^. We compared the expression of ATAD3A in A549 and A549/DDP cells and found that it was upregulated in the 549/DDP cells. The high expression of ATAD3A was related to cisplatin resistance in lung adenocarcinoma in our studies, which is in agreement with other studies where silencing ATAd3A expression reduced cisplatin resistance in prostate cancer and cisplatin, doxorubicin, and paclitaxel resistance in uterine cervical cancer^[37]^.

Mitochondria are organized in a small, fragmented or tubular, dynamic network that undergoes continuous remodeling dependent on the processes of fusion and ﬁssion^[11]^. Studies have demonstrated a dynamic imbalance of fission and fusion in cancer cells, where there is a disturbed equilibrium between fission or fusion protein activity or expression levels^[38]^. It has been reported that mitochondrial fission is frequently increased in cancer cells due to the downregulation of the mitofusin proteins including Mfn1, Mfn2, and Opa1 and/or the upregulation of Drp1^[39]^. In our study, we found that a low expression of Mfn2 was related to cisplatin resistance in lung adenocarcinoma cells, and the expression of Mfn2 in A549/DDP cells was significantly increased following co-treatment with cisplatin and SMI, followed by reduced mitochondrial fragmentation. The sensitivity to cisplatin was further confirmed by reducing the expression level of ATAD3A in A549/DDP cells co-treated with cisplatin and SMI. Mitochondrial mass and ΔΨm are important indices for evaluating mitochondrial function, and they are frequently followed by apoptosis^[40]^. In our study, we observed that mitochondrial mass and ΔΨm were decreased by co-treatment with cisplatin and SMI in A549/DDP cells, which indicated that SMI enhancement of cisplatin sensitivity was related to apoptosis. It is generally believed that continuous mitochondrial fusion can preserve cellular integrity, protect against autophagy in response to nutrient deprivation, and fission is helpful for maintaining mitochondrial health by selectively eliminating damaged mitochondrial proteins through apoptotic pathways in normal cells^[41]^. However, mitochondrial morphology, regardless of whether characterized by small and fragmented or long and interconnected tubule mitochondria, is related to the cellular processes and different circumstances the cell is under^[42]^. Furthermore, because mitochondrial function is closely related to mitochondrial dynamics, highly activated aerobic glycolysis has been linked to mitochondrial fusion and ﬁssion in many types of cancer^[43]^. Additionally, a switch of cells from one form of ATP production to another is also likely accompanied by a change in mitochondrial structure^[42]^. Previous studies have shown that cells with mitochondria that have undergone fusion rather than fission are more likely to generate ATP via oxidative phosphorylation (OXPHOS) than glycolysis. Mfn2 plays an important role in increasing the expression of OXPHOS genes^[44]^. In our study, A549/DDP cells presented with punctate and fragmented mitochondria when treated with cisplatin long term. Co-treatment with cisplatin and SMI caused mitochondria to fuse and appear as thread-like and filamentous, which was followed by Mfn2 upregulation. More powerful evidence came from our recent study showing that cisplatin-resistant A549/DDP cells were more dependent on aerobic glycolysis than cisplatin-sensitive A549 cells. In addition, co-treatment with cisplatin and SMI reduced glycolysis in A549/DDP cells with reduced expression levels of key glycolytic enzymes such as hexokinase 2 (HK2), pyruvate kinase M1/2 (PKM1/2), glucose transporter 1 (GLUT1), and pyruvate dehydrogenase (PDH)^[9]^. In summary, SMI combined with cisplatin promoted mitochondrial fusion by upregulating Mfn2 expression, which switched cisplatin-resistant cells from glycolysis to OXPHOS.

A recent study found that SMI attenuates hypoxia/reoxygenation-induced mitochondrial dysfunction and cell damage^[45]^. In our study, we found that SMI enhanced cancer cells apoptosis by regulating mitochondrial dynamics. SMI is extracted from two crude drugs, *Radix Ginseng Rubra* and *Radix Ophiopogonis *root. The main bioactive constituents of *Radix Ginseng Rubra *are the ginsenosides, and steroidal saponins are the principal active components of *Radix Ophiopogonis*^[46,47]^. Some of ginsenosides have been found to induce cell apoptosis by increasing mitochondrial membrane permeability, promote the release of cytochrome C into the cytosol, and activate caspase proteases^[48]^. Diosgenin has also been reported to induce mitochondrial apoptosis by enhancing the production of ROS and cleaved caspase-9 and regulates the balance of Bax/Bcl-2^[49]^. In this study, the mechanism of SMI combined with cisplatin inducing mitochondrial apoptosis by upregulating Mfn2 expression might be associated with the active components of *Radix Ginseng Rubra* and *Radix Ophiopogonis*.

**Figure 6:**
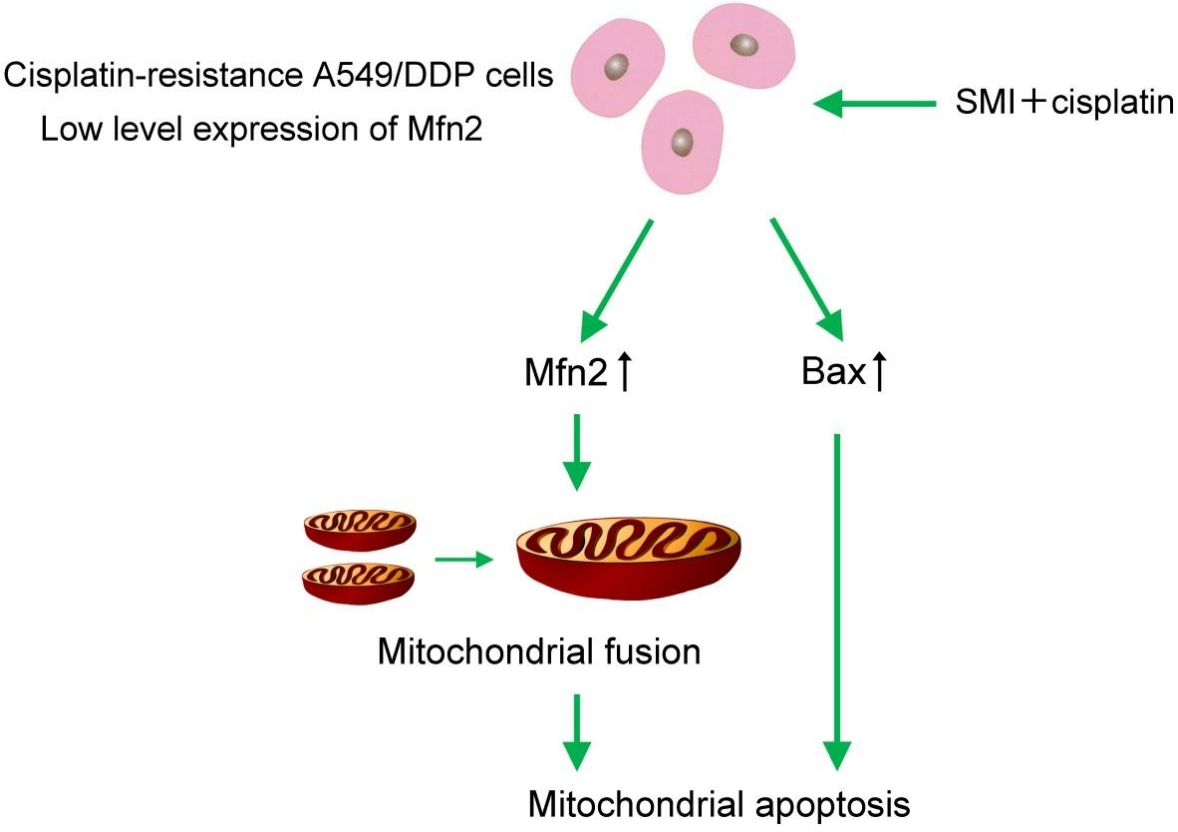
The combination of SMI and cisplatin induced apoptosis through regulation of Mfn2-dependent mitochondrial dynamics in cisplatin-resistant A549 cells. SMI: Shenmai injection.

In conclusion, we first confirmed that the expression of Mfn2 in lung adenocarcinoma was lower than that in matched paracancerous tissues. Second, we demonstrated that Mfn2 expression in cisplatin-resistant lung adenocarcinoma A549/DDP cells was lower than that in cisplatin-susceptible A549 cells. Finally, we found that co-treatment with SMI and cisplatin enhanced lung adenocarcinoma apoptosis through regulation of Mfn2-dependent mitochondrial dynamics [Figure 6]. This study is the first to focus on mitochondrial dynamics in the study of the pharmacological mechanisms of SMI for tumor therapy. These findings highlight the therapeutic potential of mitochondrial dynamics regulation as a novel strategy to overcome drug resistance in lung cancer. Furthermore, these results provide direct scientific data on SMI, a modern Chinese medicinal injection, as an effective chemotherapy sensitizer in tumor adjunctive treatment.

## DECLARATIONS

### Availability of data and materials

The data used to support the findings of this study are available from the corresponding author upon request.

### Copyright

© The Author(s) 2021.
